# Entrepreneurial fear of failure among college students: A scoping review of literature from 2010 to 2023

**DOI:** 10.1016/j.heliyon.2024.e31072

**Published:** 2024-05-11

**Authors:** Yuan Gao, Xiao Wang, Jinjin Lu, Bing Chen, Kirsty Morrin

**Affiliations:** a Academy of Future Education, Xi'an Jiaotong-Liverpool University, Soochow, China; b School of Law and Social Justice, University of Liverpool, Liverpool, UK; c International Business School Suzhou, Xi'an Jiaotong-Liverpool University, Soochow, China; d Department of Urban Planning and Design, Xi'an Jiaotong-Liverpool University, Soochow, China

**Keywords:** Fear of failure, College students, Entrepreneurship, Entrepreneurship psychology, Scoping review

## Abstract

Entrepreneurial fear of failure (EFoF) is a psychological barrier to college entrepreneurship. Current research on EFoF among college students is at an emerging stage, and relevant evidence, such as the factors influencing EFoF, remains unclear and unsystematic. Most EFoF studies treat college students as general entrepreneurs, neglecting their unique social identities and situational contexts that influence their EFoF, such as parents and education. It is essential to provide a thorough and organized review to comprehensively understand the role of the EFoF in college student entrepreneurship. A scoping review was conducted following the PRISMA-ScR protocol to offer a broad exploration and mapping of the literature. This review identified 35 studies published from 2010 to 2023 through a rigorous search and sift across five prominent databases. Descriptive and thematic analyses revealed 1) the characteristics of the included studies, 2) the exogenous and endogenous factors that influence college students' EFoF, 3) the outcomes of EFoF on college students, and 4) research gaps. By mapping and synthesizing the literature, this scoping review contributes to the theory by providing a comprehensive overview of EFoF among college students and identifying research gaps, which illuminates future research directions. The findings also offer practical insights for entrepreneurship practitioners, including college student entrepreneurs, policymakers, and educators, to better understand the role of EFoF among college students, thus effectively promoting the development of college student entrepreneurship.

## 1 Introduction

Entrepreneurship is a dynamic and ambiguous process of extracting value through innovation in which individuals identify and explore opportunities to pursue benefits while assuming various risks [1,2]. In the entrepreneurial community, college student entrepreneurs have emerged as an indispensable force driven by their distinctive attributes, such as solid knowledge reserves and innovative thinking. Countries and societies recognize the diverse benefits of college student entrepreneurship, including technological advancement and job creation, thus promoting entrepreneurial activities among college students on a large scale [[3], [4], [5]]. However, college students’ entrepreneurship is far from optimistic [3]. According to a nationwide report on China in 2021, approximately 96 % of college students had entrepreneurial ideas before, but only 14 % of these students were actively prepared for or engaged in entrepreneurial endeavors [6]. A study in the Chinese context showed that the failure rate of college student entrepreneurship was up to 95 % [6]. Therefore, numerous college students have a great passion for entrepreneurship, but simultaneously experience entrepreneurial fear of failure (EFoF), resulting in hesitation or abandonment of their entrepreneurial pursuits [7,8].

EFoF is a stable tendency to avoid failure and a psychological response triggered by failure-relevant cues within the environment [9]. It is a prominent psychological barrier that prevents college students from pursuing entrepreneurship [[10], [11], [12], [13]]. Specifically, when college students plan or practice entrepreneurship, great risks and uncertainties inevitably make them consider the driving factors and consequences of business failure [7,9]. For example, Kollmann et al. [14] found that failure-related clues, including resource shortages and insufficient liquidity, could evoke an EFoF among college students. Similarly, Chua and Bedford [8] found that some potential failure scenarios can induce EFoF in college students, including financial damage (e.g., bankruptcy), psychological damage (e.g., loss of reputation), and career damage (e.g., falling behind peers). Therefore, college students spontaneously detest and avoid the drivers and consequences related to failure, as entrepreneurial failure is still considered a stigma and a shameful experience under social norms [15,16]. Thus, EFoF exerts negative effects on college students’ entrepreneurship. Duong [17] found that EFoF weakens the transition from entrepreneurial intention to behavior among college students. EFoF can also harm college students' self-efficacy, risk perception, and entrepreneurial decision-making [18,19]. Although prior studies have recognized the adverse influence of EFoF on college students, the present exploration of EFoF among college students is at an emerging stage, and relevant evidence, including the factors influencing their EFoF, remains ambiguous and unsystematic. Therefore, it is important to present a well-organized review to gain a thorough understanding of the role of the EFoF in college student entrepreneurship.

In addition, college students are a distinct group within the realm of EFoF research, yet this aspect has been largely overlooked in previous research. Most college student entrepreneurs are in an emerging adulthood stage, gradually transitioning from adolescence to adulthood [20]. At this stage, college students mainly play the roles of students on campus and children in their families in social relationships. The introduction of entrepreneurial roles brings them new challenges and responsibilities, influencing their understanding of risks and failures [21]. This may lead to a gap between college and conventional entrepreneurs, including financial resources, interpersonal capital, and work experience [22].

For unique college entrepreneurs in their emerging adulthood [20,21], most studies have disregarded the social and cultural factors influencing their EFoF, such as education and parents. For example, Smail et al. [23] found that entrepreneurship education significantly alleviates the adverse effects of college students' fear of failure. However, parental support can mitigate the burden that EFoF imposes on college student entrepreneurs [24]. Most previous EFoF studies have regarded college students as general entrepreneurs, neglecting the unique sociocultural elements that shape their EFoF experiences. Therefore, the factors influencing EFoF identified among general entrepreneurs may not align with the psychological traits of college students, thereby failing to interpret the role of EFoF among college students. This gap further echoes the rationale and research objectives of this study. In EFoF research, we need to focus on a specific group of college students, together with their identities and contexts. By reviewing, mapping, and synthesizing the related evidence, our research provides an appropriate and comprehensive understanding of college students' EFoF. To achieve this objective, we adopted a scoping review paradigm by comprehensively gathering and analyzing pertinent studies on EFoF among college students from 2010 to 2023. Based on these gaps, we propose the following research questions.

1. What are the key findings of studies on EFoF among college students?
2. Based on the results of Research Question 1, which aspects of EFoF among college students should be explored in the future?

This study makes diverse theoretical and practical contributions. First, it contributes to the EFoF literature by focusing exclusively on college students. College students are a large and unique part of the entrepreneurial community, and experience special identities and contexts during their emerging adulthood. Previous studies have overlooked this gap, making it difficult to explain the role of college students’ EFoF comprehensively and accurately. We employed a scoping review to fill this gap and reveal the diverse aspects of the EFoF phenomenon in college students, such as the characteristics of previous research, factors influencing college students' EFoF, and its impacts on psychology and behavior. This scoping review contributes to the theoretical knowledge and development of EFoF and college student entrepreneurship. Second, our scoping review critically analyzed the included literature and identified significant gaps in the current research on EFoF among college students. Addressing these crucial issues, such as inconsistent measurement tools and the absence of sociocultural factors (e.g., parental influence), is a pivotal step in advancing college students' EFoF research. Future studies could draw inspiration from these findings, thereby establishing a more comprehensive theoretical framework with enriched perspectives on the EFoF among college students.

Third, this scoping review offers practical implications for various stakeholders involved in college student entrepreneurship. College student entrepreneurship plays an irreplaceable role in promoting economic development and technological innovation and tackling employment issues [[3], [4], [5]]. However, EFoF remains a significant mental obstacle for college students starting new businesses [7]. Therefore, this research holds particular urgency for college students harboring entrepreneurial ambitions. It offers them a thorough understanding of EFoF, which is a vital step in fostering a resilient and innovative entrepreneurial spirit. Besides, various stakeholders in college student entrepreneurship can acquire crucial insights from this study, thereby assisting the next generation of entrepreneurs in navigating their psychological barrier EFoF in their entrepreneurial journey. For instance, the entrepreneurial education and training provided by higher education could include knowledge of entrepreneurial psychology (e.g., EFoF) to aid college student entrepreneurs in overcoming EFoF. Simultaneously, the government should offer support systems and consulting services to help establish a balanced mindset, thus contributing to the effective development of college students’ entrepreneurship. Such collaborative efforts among educational institutions, government bodies, and other stakeholders are essential for cultivating a robust entrepreneurial ecosystem that nurtures the growth and resilience of future college student entrepreneurs.

## 2 Methods

A scoping review is a literature review paradigm that systematically identifies, maps, and summarizes existing research on a broad topic, providing a comprehensive overview of the key concepts, evidence, and gaps in the literature [25,26]. The rationale for adopting a scoping paradigm has three aspects. First, scoping reviews are suitable for broad and exploratory research questions [26,27]. Unlike systematic reviews, which address specific and precise research questions, scoping reviews are acceptable when the objective is to explore the extent and nature of the literature within a broader thematic area. This aligns well with the broad nature of our research, as the scoping review allows for a preliminary assessment of existing research and the identification of a diverse array of studies across different contexts and methodologies. Given the nascent state of research on EFoF among college students and the dispersed nature of existing evidence, a scoping review serves as an essential step. It enables us to collect and synthesize different evidence, offering a clear overview of the current state of the literature on college students' EFoF, thus laying the groundwork for more detailed and focused inquiries in the future.

Second, scoping reviews are well suited for investigating emerging research topics. Current research on EFoF among college students is in an emerging and evolving stage, characterized by evidence that is both widely dispersed and varied in nature (e.g., empirical and theoretical). This diversity necessitates a scoping review paradigm with a flexible and broad investigation approach. It enables us with a thorough exploration, mapping, and discussion of the topic across a range of evidence types, which is crucial for delineating the current knowledge landscape, identifying gaps, and directing future research on EFoF among college students. By contrast, the narrow focus and strict selection criteria of systematic reviews might not be appropriate for our topic, as it may overlook the varied evidence critical for fully grasping this emerging area of study [25,26].

Third, scoping reviews offer advantages in terms of iterations, flexibility, and inclusivity. Emerging research topics like EFoF among college students are often in the developmental phase, often characterized by unclearly defined research fields and evidence scattered across interdisciplinary databases. Scoping reviews can address these deviations by adjusting the protocol, including the inclusion and exclusion criteria, which offers remarkable flexibility and iteration [28,29]. This methodology also allows the incorporation of different studies with varying research designs, methods, and types of evidence [26,29]. Its inclusivity enables the development of a comprehensive literature map and future insights into college students' EFoF [25,26]. By contrast, a systematic review, with its predefined and rigorous methodological process, is not suitable for addressing the exploratory nature of emerging topics, as it typically focuses on reviewing a standardized body of literature for specific and clearly defined research questions and issues [25].

Therefore, we adopted a scoping review paradigm to provide an overarching view of college students' EFoF by synthesizing, presenting, and discussing various studies, thereby offering valuable insights for future research and entrepreneurship practitioners. Following the methodological framework of Arksey and O'Malley [28] and Levac et al. [29], the scoping review contained five main parts: 1) determining the research design and questions, 2) searching for relevant studies, 3) screening relevant studies, 4) charting the data, and 5) collating, summarizing, and reporting the findings.

### 2.1 Relevant studies identification

Following Arksey and O'Malley's [28] scoping review protocol, we preliminarily developed a search strategy to identify relevant studies. To ensure precision and relevance in our search [28], we contacted a librarian and an information specialist from the university to assist us in identifying search terms and appropriate databases. We initially deliberated on the search terms, given that fear of failure is an established psychological construct extensively utilized across different disciplines, including entrepreneurship [7]. The librarian also reminded us that “Atychiphobia” is another term replacing fear of failure, derived from the Greek words “atyche” and “phobos.” We were also advised to apply asterisk wildcards to entrepreneur (entrepreneur*), as it allows for different forms of terms related to entrepreneurship, including entrepreneurship, entrepreneurial, and entrepreneurism. The final search terms included three dimensions: 1) “fear of failure” OR “fear of failing” OR “atychiphobia”; 2) entrepreneur* (including “entrepreneurial,” “entrepreneurship,” etc.), and 3) “college” OR “university” OR “student.” Table 1 lists the search strings generated by combining keywords with Boolean OR and AND operators.

**Table 1:** Database search strings.

**Database**	**Boolean/Phrase:**	**N** =
Web of Science Core Collection	((TS=(“fear of failure” OR “fear of failing” OR “atychiphobia")) AND TS=(entrepreneur*)) AND ALL=(“college” or “university” or “student")	32
Scopus	(TITLE-ABS-KEY (“fear of failure” OR “fear of failing” OR “atychiphobia”) AND TITLE-ABS-KEY (entrepreneur*) AND ALL (“college” OR “university” OR “student”))	239
Emerald Insight	(content-type:article) AND (abstract:"fear of failure” AND (abstract:"entrepreneur*") AND (“college” OR “University” OR “student”))	41
Business Source Ultimate	AB (fear of failure OR AB fear of failing OR AB atychiphobia) AND AB entrepreneur* AND TX (college OR university OR student)	152
ABI/INFORM (ProQuest)	abstract(fear of failure OR fear of failing OR atychiphobia) AND abstract(entrepreneur*) AND (college or university or student)	155

The researchers also discussed with an information specialist to select suitable databases that would offer extensive coverage in relevant multidisciplinary fields, such as business and psychology. This step is vital to align with Arksey and O'Malley's [28] principle of broad coverage in scope reviews. After a thorough discussion, we selected five globally-recognized and research-focused databases that provide multidisciplinary and comprehensive resources. The five databases are Web of Science Core Collection, Scopus, Emerald Insight, Business Source Ultimate, and ABI/Inform (ProQuest). This selection ensured that our literature search was as inclusive and representative as possible of the existing body of work on EFoF among college students. The final search was completed on February 1, 2024, and 619 initial results were obtained. These were then meticulously screened in Excel for relevance and quality before further analysis.

### 2.2 Study screening and selection

**Fig. 1:**
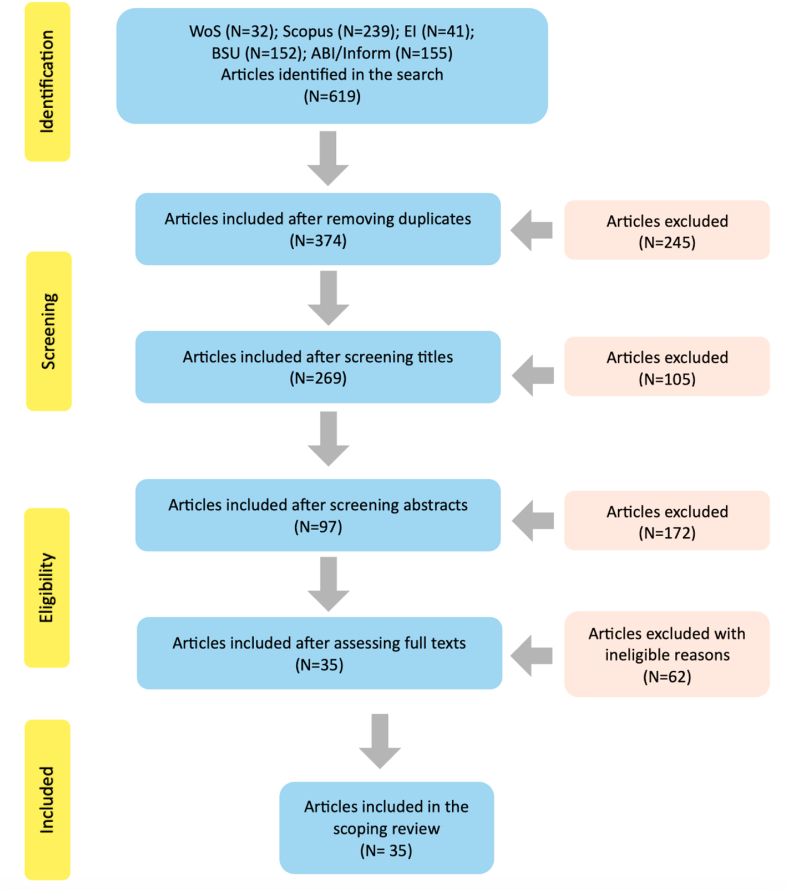
PRSIMA-ScR flow diagram of the study selection process.

According to the PRISMA-ScR (Systematic Reviews and Meta-Analyses extension for Scoping Reviews) paradigm [30], we discussed and developed a rigorous data filtering procedure to ensure the replicability of the study. Fig. 1 illustrates the study selection process, which includes searching databases, screening titles and abstracts, screening full-text articles, and selecting articles that meet the inclusion criteria. In the preliminary phase, 619 research items were identified from five databases. After preliminary data screening, 245 duplicate records were removed, resulting in 374 distinct research entries for further scrutiny and evaluation.

The scoping review used the following five inclusion and exclusion criteria: First, the included studies discussed the fear of failure in the entrepreneurship domain. Second, the included studies focused on a cohort of college students. Third, to ensure literature quality, our selection criteria were restricted to peer-reviewed journal articles, thereby excluding conference proceedings, book chapters, and gray literature sources such as news reports. The selected journals were indexed using prestigious retrieval platforms, including the Social Science Citation Index and Journal Citation Reports. Fourth, the timeframe of this scoping review was 2010–2023. During the literature identification process, we discovered that research on college students' EFoF emerged around 2010 [[31], [32], [33]], which was attributed to the popularity of studying the fear of failure in entrepreneurship. Until now, the 13-year research period has effectively reflected the developmental trends and state of this topic. Fifth, the scoping review included only studies published in English.

After removing the 245 duplicate entries, we applied the criteria above to retrieve titles and abstracts from 374 different papers. Following title screening, 105 articles were excluded, and this was followed by excluding 172 articles after abstract screening due to irrelevance or failure to meet the specified criteria, with 97 articles remaining. Although the titles and abstracts of these 97 articles met our inclusion criteria, their full-text content may not fully align with our review scope, regarding the entrepreneurial theme, participant information, or other relevant aspects related to college students' EFoF. To address eligibility concerns, the research team thoroughly evaluated the full texts of these 97 articles. Any discrepancies encountered during this process were resolved through collaborative group discussions among team members. After rigorous examination, screening, and comprehensive discussion, 35 articles were selected for the subsequent scoping review.

### 2.3 Data charting, collating, and results reporting

Following the paradigm suggested by Arksey and O'Malley [28], we developed a data chart form, as shown in the supplementary material file. Critical information was extracted from 35 studies, including the author(s), year of publication, research objective, perspective on EFoF, research design, participants' knowledge, and main findings.

Utilizing this structured approach enabled us to systematically analyze and synthesize the findings of our scoping review to directly address our research questions. Through this organized method, we were able to outline the main characteristics of current EFoF research among college students, underscoring key themes and findings. Furthermore, our analysis identified research gaps, paving the way for future investigations into EFoF within this group.

## 3 Results

The scoping review included 35 articles (Fig. 1). The results are presented in terms of three main aspects. First, the relevant descriptive indicators from the included studies were summarized to illustrate the basic characteristics of the research on college students' EFoF. The indicators include demographic information, participant characteristics, and instruments used to measure the EFoF.

Second, several crucial themes were extracted to identify the factors that primarily affect college students' EFoF and the impact of EFoF on them. Third, based on the findings and limitations of previous research, we encapsulated pivotal gaps to provide theoretical insights and practical significance for future research.

### 3.1 Descriptive analysis of the included studies

#### 3.1.1 Demographic information

From the pool of 35 articles, the researchers reported demographic information, as illustrated in Table 2. It encompasses the frequency of publications every five years, distribution of research locations, and methodological paradigms employed. Regarding the year of publication, the number of studies on EFoF among college students is constantly increasing. This can be attributed to two factors. First, the emergence of college student entrepreneurship is closely related to economic growth and the increasing prevalence of entrepreneurial education in tertiary schools [34]. Second, some researchers have gradually recognized the distinctiveness of college students owing to their social identities and situational contexts [8,10], which underscores the importance of future research addressing this topic.

**Table 2:** Demographic information (N = 35).

Demographics	N =	%
**Year of Publication**		
2010–2015	8	22.9
2015–2020	12	34.3
2020–2023	15	42.8
**Distribution of Regions**		
Asia	19	51.4
Europe	9	24.3
North America	3	8.1
South America	3	8.1
Oceania	1	2.7
Africa	2	5.4
**Methodological Paradigm**		
Quantitative methods	30	85.7
Qualitative methods	3	8.6
Mixed methods	2	5.7

We conducted a thorough count of the locations of the included studies and categorized multinational studies [12] based on different continents. The articles in this scoping review covered 33 countries and regions. As shown in Table 2, the current EFoF research on college students is mainly distributed in Asia (N = 19), followed by Europe (N = 9). Specifically, China (N = 4) and India (N = 3) were the most studied locations on this topic [10,33,35,36]. We believe that this distribution is consistent with the current global economic landscape because China and India have robust economic growth, large populations, and substantial entrepreneurial activities. In addition, this distribution reflects the varying emphasis that countries and regions place on college student entrepreneurship, warranting further research.

Regarding the methodological paradigm, the majority of studies employed quantitative methods (N = 30). These studies mostly utilized statistical techniques such as structural equation modeling to examine the different roles of the EFoF (e.g., mediators and moderators) [17,18,37,38]. In comparison, a limited number of studies (N = 3) opted for qualitative methods, and only two studies utilized a mixed-methods design to explore the status of EFoF within the college student cohort [31,39]. Although numerous quantitative studies have yielded significant findings elucidating the relationship between the EFoF and other variables, the evidence remains fragmented and lacks an in-depth explanation. Therefore, future research should incorporate diverse qualitative methods and offer additional insights based on quantitative findings. This will help to explore the intricate internal mechanisms underlying college students’ EFoF.

#### 3.1.2 Portrait of respondents

The respondents in the included articles were all college students, which was one of our screening criteria. Most college students were selected as research respondents because they shared commonalities on a particular feature. For instance, many studies selected college students with backgrounds in business, economics, or entrepreneurship education as samples [12,37,40]. Because students in these majors and courses tend to display more evident entrepreneurial intentions and characteristics, they are often regarded as emerging entrepreneurs. This review also identified studies that investigated gender differences among college student entrepreneurs. Meeralam and Adeinat [38] focused on female students in Saudi Arabia as their study samples. It was motivated by the recognition that college students played a significant role in entrepreneurship, but female students might encounter additional business challenges and a higher risk of failure due to the “entrenched gender bias” in their region [38] (p. 392).

The respondents were situated at different stages of their entrepreneurial journey. Most college students are potential entrepreneurs with entrepreneurial intentions, but have yet to undertake any practices [8,10]. A few are university postgraduate students with employment and entrepreneurial experiences [17,32].

Although these samples share the identities of college students, they can still manifest varying characteristics owing to contextual differences, potentially resulting in variations in the EFoF. This aspect has been overlooked in studies that have solely used college students as samples. Future research on EFoF should consider this point and explore the differences in EFoF among different groups of college student entrepreneurs.

#### 3.1.3 Instruments for measuring EFoF

Drawing on 30 quantitative studies, this scoping review also summarizes the instruments for measuring EFoF among college students. Existing measurement tools are categorized into three types: single-dimensional, multidimensional, and hybrid. The most prevalent method is a single-dimensional tool with a dichotomous question (also known as a closed dummy question) that asks participants whether they would give up entrepreneurial activities due to fear of failure. The question was adapted from the Global Entrepreneurship Monitor (GEM), a global project investigating the degree of EFoF in different countries and regions, by asking the following question: “Would fear of failure prevent you from starting a business?” [41]. Although many EFoF studies have adopted this single-question measurement [[42], [43], [44]], researchers have criticized its simplicity and neglect of the multidimensional essence of EFoF [7].

The multidimensional approaches are gaining popularity in EFoF studies, leading to the development of various instruments. The two most recognized are the Performance Failure Inventory Appraisal (PFAI) by Conroy et al. [45] and the Entrepreneurial Fear of Failure Scale by Cacciotti et al. [9]. However, the PFAI is applied to various achievement scenarios, such as competitive sports and exams, and not exclusively to entrepreneurial activities. Cacciotti, with the research team, developed a specialized scale with seven dimensions and 21 items for the experience of failure in the entrepreneurial context, but it was not exclusively designed for college students. Future researchers can consider developing or modifying measurement tools suitable for the college students' EFoF.

Some studies have adopted, modified, or merged scales to measure EFoF among college students [38,46], such as the Achievement Motivates Scale by Lang and Fries [47]. Even fewer studies have adopted hybrid measurements, which activate college students' inner feelings by simulating scenarios (typically texts, pictures, or videos) and subsequently ask them to rate the intensity of their EFoF [33]. However, this method still requires further empirical testing of its reliability and validity in different contexts.

### 3.2 Factors influencing EFoF among college students

**Fig. 2:**
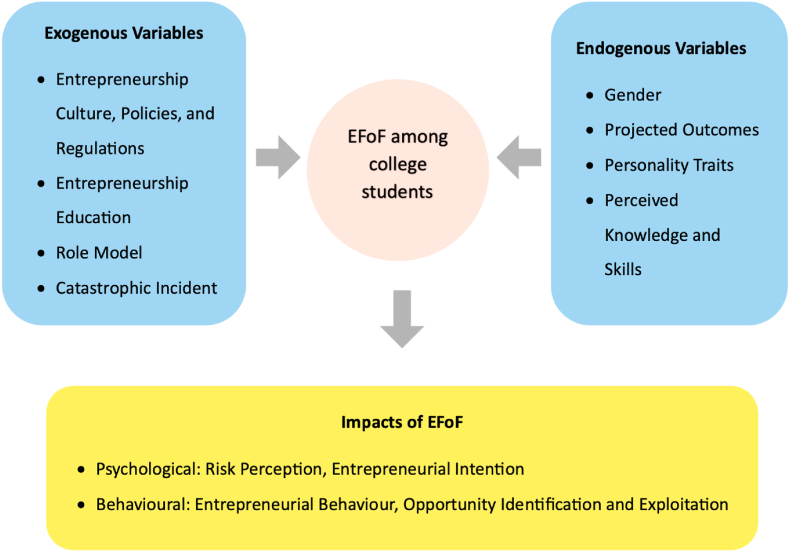
The influence factors and outcomes of EFoF among college students.

In the following sections, we conduct a critical review of the EFoF literature to identify the factors that may influence college students' EFoF, as depicted in Fig. 2. We adopted the person–environment framework of EFoF proposed by Cacciotti and Hayton [48]. As the framework highlights an individual's subjective experience and contextualization of EFoF, we categorized the identified factors into environmental and individual levels. Environmental-level elements were designated as exogenous variables, encompassing *Entrepreneurship Culture, Policies and Regulations*, *Entrepreneurship Education*, *Role Model*, and *Catastrophic Incident*. Similarly, individual-level elements are referred to as endogenous variables and include *Gender*, *Projected Outcomes*, *Personality Traits*, and *Perceived Knowledge and Skills*.

#### 3.2.1 Exogenous variables

**Entrepreneurship Culture, Policies, and Regulations.** Institutional factors, including entrepreneurship culture, policies, and regulations, shape the context in which entrepreneurship occurs [49]. Culture can significantly influence entrepreneurship by cultivating societal norms and individual values pertaining to risk-taking, entrepreneurial legitimacy, tolerance of failure, etc. [44,50,51]. For instance, cross-cultural analysis has shown that national culture influences the perceptions of entrepreneurial barriers and EFoF among college students [12]. Chua and Bedford [8] also confirmed a similar finding, suggesting that face-saving culture (known as *Mianzi* in Mandarin) is a crucial concern for college student entrepreneurs in collectivist Asian countries such as China and Singapore, leading them to exhibit higher levels of EFoF than their counterparts in Western countries [8].

Moreover, entrepreneurship policies and regulations are crucial external factors established by authorities to govern and facilitate entrepreneurial activities [52]. This scoping review reveals that many studies have examined the influence of entrepreneurship policies and regulations on college student cohorts. In these studies, scholars frequently employed their findings to advocate for the enhancement of entrepreneurship policies and regulations by governments and relevant departments to alleviate the adverse effects of EFoF on college student entrepreneurs [37,38,49,53]. However, Urbano et al. [54] offer contrary findings, noting that economic freedom developed through governance can alleviate the negative impacts of EFoF in developed countries, but not in developing countries. We consider this result to be attributable to differences in corruption between developed and developing countries that affect individual EFoF [55]; however, further research is required.

**Entrepreneurship Education**. As an exogenous factor, entrepreneurship education plays a vital role in influencing college students' entrepreneurial attitudes and perceptions, especially EFoF. Duong and Vu [18] conducted research on 1890 college students in Vietnam and found that EFoF exerted negative moderating effects on entrepreneurial self-efficacy and intention. Smail et al. [23] investigated the determinants of entrepreneurial risk and found that entrepreneurship education could significantly attenuate college students’ EFoF in the United Arab Emirates. Entrepreneurship education can provide college students with business knowledge, skills, and resources, thus promoting their intentions and self-efficacy [56,57]. Students may have gained a more precise and positive understanding of the entrepreneurial risks and failures that mitigated their EFoF [23,36,39]. Numerous articles have advocated for the expansion and enhancement of entrepreneurship education with the aim of ameliorating the adverse impacts of EFoF on college students [8,10,17,24,37]. Nevertheless, some studies indicate that entrepreneurship education dampens the entrepreneurial intentions of certain college students as it preemptively exposes too much negative information, such as uncertainty and bankruptcy [58]. This implies that entrepreneurship education may heighten the EFoF for this specific group, although validation is still lacking.

**Role Model**. Note that business role models, as exogenous factors, can exert certain impacts on college students' EFoF. Studies suggest that business role models could play a constructive role in shaping the attitudes of prospective college student entrepreneurs by portraying favorable depictions of entrepreneurship and offering valuable insights into entrepreneurial processes and skills [10]. These positive influences could help mitigate college students' perceptions of the probability of business failure, thereby alleviating the EFoF [53,59]. As the number of entrepreneurial role models increases, the social acceptance of entrepreneurship gradually increases, which legitimizes entrepreneurship and reduces students' EFoF [60]. However, Wyrwich et al. [60] also found that failed entrepreneurs can amplify college students' EFoF.

**Catastrophic Incident**. A catastrophic incident refers to an unexpected event or crisis that occurs on a large scale and affects the public, often with far-reaching consequences and unforeseen impacts on various aspects of society, including health, economy, and daily life [61]. Given the sporadic nature of catastrophic incidents, few studies have addressed the influence of public announcement events on entrepreneurial EFoF. Notably, Sheng and Chen [46] found that the severity of the COVID-19 pandemic positively influenced the entrepreneurial inclination of Chinese college students, and that this relationship was further mediated by regulatory focus and EFoF. These catastrophic incidents tend to generate an unstable environment with ambiguity, which may intensify the perceived risks, heighten apprehension, and result in a greater EFoF, prompting college students to approach entrepreneurial ventures with caution and hesitation [46,62].

#### 3.2.2 Endogenous variables

**Gender**. Many entrepreneurship studies have considered gender a crucial demographic indicator, and research has identified differences in the manifestation of EFoF among college students of different genders. A study conducted in the United States found that the individuals identifying as feminine exhibited a higher level of EFoF than those identifying as masculine, resulting in lower entrepreneurial intentions [63]. An investigation in Vietnam indicated differences in the mediating influence of EFoF on the relationship between entrepreneurial education and intentions among male and female students [18]. Similar findings were supported in a Spanish university where female students demonstrated greater EFoF than male students because they were possibly more pessimistic and concerned about their business capabilities and potential barriers [31]. However, Shinnar et al. [12] found that gender differences in EFoF apply to college students in America and Belgium, but not in China. The gender differences observed in EFoF may be related to social norms, gender division of labor, etc. [64], warranting an in-depth investigation in the future.

**Projected Outcomes.** According to the cognitive appraisal theory, potential college student entrepreneurs assess ambient opportunities, resources, and risks when considering starting a new business [65]. If they evaluate these difficulties and challenges as being highly related to business or project failure, the projected failure outcomes will trigger their EFoF. This viewpoint is supported by relevant research. Using a sample of German university students, Kollmann et al. [14] found that EFoF can be activated by failure-related cues in the business environment. These clues were closely related to resources, markets, and social capital, such as “insufficient liquidity or lack of financial resources,” “loss of customer demand,” and “break up with co-founders” [14]. Chua and Bedford [8] summarized the factors that directly trigger EFoF by asking college students to imagine and discuss entrepreneurial failure scenarios. The factors include financial, psychological, and career damage, such as bankruptcy, losing face, disappointing others, and falling behind peers. Unlike Kollmann's [14] focus on the causes of failure, these pertinent factors stem from the consequences of failure, which can elicit college students' EFoF.

**Personal Traits.** Personal traits shape an entrepreneur's mindset, behaviors, and capabilities toward opportunities and risks. Some studies have found that college students with different personal traits may exhibit varying degrees of EFoF. Ukil and Jenkins [66] found that, although EFoF undermined college students' entrepreneurial intentions, their resilient trait reversely reduced EFoF, thereby alleviating the adverse harm it caused. Martins et al. [13] reported similar findings regarding the relationship between college students' confidence traits and the EFoF.

**Perceived Knowledge and Skills**. The subjective assessment of an individual's understanding and proficiency in entrepreneurial concepts, strategies, and practices is referred to as perceived knowledge and skills in entrepreneurship. The studies included in this research consistently demonstrate that college students' perceived knowledge and skills can influence their entrepreneurial intentions, behavior, and EFoF [13,38]. For instance, Ekore and Okekeocha [67] found that perceived entrepreneurial capacity accurately predicted EFoF among 1100 college students in Nigeria. Similarly, Belwal et al. [39] discovered that college students in Oman exhibit strong entrepreneurial intentions but lack professional knowledge and skills. Consequently, students' high EFoF became a barrier that deterred them from pursuing entrepreneurship.

### 3.3 Impacts of EFoF on college students

According to the included articles, the impacts of EFoF on college students are generally regarded as psychological and behavioral outcomes. When college students receive entrepreneurial information, they undergo an evaluation process involving opportunities, capabilities, resources, and risks. If the evaluation results are associated with business failure, we expect EFoF to be triggered, which subsequently influences various psychological and behavioral issues, such as entrepreneurial intentions, attitudes, emotions, and decision-making [7,8].

#### 3.3.1 Psychological outcomes

One of the most significant psychological outcomes is that EFoF can create psychological barriers for college students, thereby hindering their entrepreneurial intentions. For example, Sousa-Filho et al. [19] found that EFoF significantly decreased entrepreneurial intention among 979 college students in businesses in Latin American countries (Brazil, Peru, Columbia, and Mexico). These outcomes have been consistently verified in different regions, grades, and genders in college student cohorts [10,24,32,35,49,65,68,69]. When college students evaluate entrepreneurship risks, they inevitably evaluate business risks and consider business failure scenarios. These unexpected scenarios include the causes (e.g., insufficient finances, uncompetitive products, and services) and consequences (e.g., bankruptcy and reputational harm) of business failure. These negative scenarios trigger EFoF, whereby college students' entrepreneurial intentions and ideas are dampened.

Some studies have analyzed the psychological outcomes of EFoF from other perspectives. Villanueva and Martins [68] suggested that EFoF had a proximal impact on college students' risk perception and assessment and subsequently affected their entrepreneurial intentions. Ng and Jenkins [37] found that EFoF alleviates the positive effects of college students' self-efficacy on entrepreneurial intention. Duong and Vu [18] similarly claimed that EFoF plays a negative role in the relationship between entrepreneurial education, self-efficacy, and college students' intentions.

#### 3.3.2 Behavioral outcomes

The impact of EFoF on the behavioral outcomes of college students is significantly reflected in its hindrance of entrepreneurial behaviors [49,70]. As an emotional component of avoidance, EFoF projects negative failure scenarios onto college students, leading them to perceive business success as a great challenge. During this process, increased EFoF may encourage college students to choose safer moves or even retreat to avoid risks and potential failure. Duong [17] and Kong et al. [10] indicated that EFoF negatively moderates the transition from entrepreneurial intentions to actions among college students. Li [33] has also claimed that EFoF affects college students' identification and exploitation of opportunities, thereby interfering with business venturing. Another study in Latin America suggested that EFoF can also impact perceived behavioral control among college students, further influencing their entrepreneurial behavior [68].

Conversely, a few studies have argued that EFoF can also exert a motivational influence on individuals' entrepreneurial behavior [7,48], but this claim has not been examined among college students. In psychology, the achievement motivation theory suggests that individuals make proactive efforts to achieve goals or standards that are threatened [71]. This theory may explain why EFoF can not only intensify individuals' perception of threats but also trigger their coping mechanisms, leading to an increase in entrepreneurial efforts. Similar findings were discovered for nascent Swedish entrepreneurs in the dairy sector and entrepreneurial managers in Mexico [72], but not among college students. Future research should investigate this phenomenon to enrich our knowledge of college students' EFoF.

### 3.4 Research gaps of EFoF among college students

#### 3.4.1 Neglected aspects for EFoF of college students

Through a scoping review, we identified a research gap in which existing studies have overlooked the distinctive attributes of college students and their specific circumstances when encountering EFoF. College students are a typical cohort in entrepreneurship practice and research, and their proportion has gradually increased due to the prevalence of entrepreneurial education and training in higher education [10,73]. However, their attitudes and experiences toward entrepreneurship are inconsistent with those of other entrepreneurs at different life stages, including their EFoF.

For instance, we know that parental interference influences the career development of college students, including risky and uncertain entrepreneurship [8,24,74]. Turulja et al. [24] have noted that informal support—mostly parental—could moderate the negative impacts of college students' EFoF on their entrepreneurial intentions. Chua and Bedford [8] (p. 331), similarly found that parents “play a larger role” in college students' EFoF in terms of financial aspects. However, no study has directly examined the role of parents in exploring their association with EFoF among college students, such as in the context of parents opposing their ongoing children engaging in entrepreneurship [6].

Many EFoF studies regard business college students as potential nascent entrepreneurs [14,33,75,76]. However, assuming that studying business or economics necessarily indicates strong entrepreneurial tendencies among college students could be indicative of a bias. Multiple factors of college experience, such as internships and extracurricular activities, likely contribute to entrepreneurial tendencies [77,78], while the university major may only be one facet. This finding echoes our suggestion that the current research lacks an exploration toward college students’ entrepreneurship, thus neglecting their unique understanding of EFoF.

#### 3.4.2 Mechanism of EFoF among college students

Another research gap pertains to the nontransparent and ambiguous mechanisms underlying EFoF among college students. We've observed that recent studies from 2022 to 2023 prefer quantitative techniques to provide evidence of possible links between college students' EFoF and proximal factors, such as entrepreneurial intentions, behaviors, self-efficacy, and passion. However, this evidence remains scattered and incomplete, calling for future researchers to consolidate these insights to construct a comprehensive mechanism of EFoF among college students. Meanwhile, few studies have proposed the dualistic impacts of EFoF on individuals, as it not only exerts inhibitory effects but also stimulates individuals' motivation and spirit of entrepreneurship [79]. This explains why EFoF can stimulate some individuals' coping mechanisms, such as self-compassion and confidence rebuilding, which drive college students to avoid failure and proactively strive for business success [7]. However, the positive effects of EFoF have not been thoroughly investigated among college students, which provides directions for future research.

In addition, the research included in this scoping review was cross-sectional in nature, focusing only on EFoF traits displayed by college students at a specific point in time. However, the EFoF is influenced by both individual experiences and changes in the external environment, implying a dynamic person–environment model [48]. Thus, cross-sectional data fail to provide a comprehensive and analytical mechanism for EFoF that could effectively explain its underlying causality and nonlinear aspects [17,35,36,79]. There is a lack of sufficient longitudinal research to establish a precise time sequence between predictors and outcomes, which is crucial for a more comprehensive understanding of the EFoF mechanism in college student cohort [17,36].

#### 3.4.3 Measurement of EFoF among college students

We observed a lack of standardized measurement tools for assessing EFoF among college students, with a diversity of approaches employed in extant studies. Table 3 lists the three main available measurement methods: single-dimensional, multidimensional, and hybrid.

**Table 3:** The summary of measurement instruments.

Measurement		Description	Examples
Single-dimensional	One dichotomous question for measuring fear of failure	Derived from and similar to the GEM question, researchers use one dummy question to ask whether participants would give up entrepreneurship due to fearing failure.	Kong et al. [10],
			Martins et al. [13],
			Urbano et al. [49]
Multidimensional	Performance Failure Appraisal Inventory (PFAI)	Developed by Conroy et al. [45], the scale consists of 5 dimensions, with a short version of 5 item questions or long version of 25 item questions.	Ng and Jenkins [37],
			Ukil and Jenkins [66]
	Entrepreneurial Fear of Failure Scale	Developed by Cacciotti et al. [9], the scale consists of 7 dimensions and 21 item questions.	Duong [17],
			Hassan et al. [35]
	Revised Achievement Motives Scale (AMS)	Developed by Lang and Fries [47], the scale consists of 10 item questions to measure hope of success and fear of failure.	Kollmann et al. [14],
			Sheng and Chen [46]
	Self-Modified Scale	Adapt and merge some existing scales to measure EFoF.	Singh Sandhu et al. [32],
			Meeralam and Adeinat [38]
Hybrid	Simulation Heuristic Measurement	The mixed approach would present a decision scenario (text) to activate participants' mindset, and require them to rate the intensity of EFoF.	Li [33]

A single-dimensional approach with one item has raised concerns among researchers [9,10,40]. Entrepreneurship is a complex process; relying on a single item is insufficient to capture the diverse responses of college students to EFoF, which limits the ability of researchers to explore the intricate relationships between EFoF and other variables. The logic of this one-item question implies negative and inhibitory effects of the EFoF, but EFoF may also have a motivational impact [7,72,80]. By neglecting this intriguing finding, the one-item question could be prone to a measurement bias.

However, the multidimensional scales are not sufficiently convergent. Most were adapted from scales for a more general scenario or cohort of people [23,46,81], such as Conroy's PFAI tool [45] and Cacciotti et al.’s EFoF scale [9], rather than precisely aiming at college students. Based on the unique characteristics of college students, the EFoF measurement tool can be further adapted to incorporate potential predictors such as parental roles.

#### 3.4.4 Coping strategies for college students handling EFoF

The studies included in this review recognize that EFoF is a barrier to college students' entrepreneurship, but no research has developed a pragmatic strategy or program to help college students confront it. Few studies do offer insights into and potential avenues for addressing this issue in the future. For instance, entrepreneurship education should focus on students' entrepreneurial psychology, in addition to lecturing on knowledge and skills. It is vital for related courses to incorporate a thorough examination of EFoF to foster a deeper understanding and resilience among college students, thereby mitigating the negative impacts associated with EFoF [13,37,38,49]. Laws and regulations should also ameliorate social norms to increase public tolerance of business failure and alleviate the prejudice caused by EFoF. Collaboration between the government and schools can facilitate financial support and an “entrepreneurship insurance system” to assist college student entrepreneurs in addressing the EFoF [10,17,35].

These suggestions are reasonable given the increasing importance of the roles of education and policy. However, research must provide pragmatic and viable projects or programs at an individual level. Engel et al. [82] used interventions and controlled experiments to offer a loving-kindness meditation model, which made it an implementable method for entrepreneurs suffering from EFoF. Further research should integrate college students’ attributes and employ individual self-regulation mechanisms to develop professional development programs that assist students in handling the adverse effects of EFoF.

## 4 Discussion

Entrepreneurship has gradually become a popular career path among college students, and EFoF is a prominent psychological factor hindering new business ventures [83]. Although researchers have extensively investigated EFoF in entrepreneurial contexts, relevant studies on college student cohorts are still emerging and scattered. This article presents a scoping review to comprehensively map and summarize the demographics and key findings on EFoF among college students to identify gaps and directions for further investigation. Table 4 summarizes the key themes influencing EFoF among college students along with crucial literature.

**Table 4:** The influencing factors on college students’ EFoF and key literature.

Themes	Key Literature
**Exogenous Factors**	
Entrepreneurship Culture, Policies, and Regulations	[12,42,55,[84], [85], [86]]
Entrepreneurship Education	[87,57,[88], [89], [90], [91], [92]]
Role Model	[42,87,60,91]
Catastrophic Incident	[46,62]
**Endogenous Factors**	
Gender	[41,64,[93], [94], [95], [96], [97]]
Projected Outcomes	[7,8,48,98]
Personality Traits	[79,99,100,101]
Perceived Knowledge and Skills	[39,43,82,89,100]

**Descriptive Summary.** The descriptive findings indicate that research on EFoF among college students is becoming increasingly significant but remains in the preliminary exploration phase. Literature has shown a steady growth since 2010, with a notable surge from 2022 onwards. This trend generally reflects three research phenomena. First, studies have intensified their attention and reaffirmed the negative impact of EFoF on college student entrepreneurship across different groups and contexts. Second, the recent surge in literature since 2022 shows a preference for the quantitative paradigm to explore the possible links between college students’ EFoF and proximal factors [17,18,35,36,38,40,46,66]. For instance, the research by Duong [17], and Meeralam and Adeinat [38] consistently highlights EFoF as a significant psychological barrier affecting various aspects of entrepreneurship, such as intentions, behaviors, risk assessment, and resilience. These studies offer substantial evidence at the initial research stage of EFoF in college students, and urge future researchers to develop a theoretical framework for EFoF among this group. Third, some studies have broadened their investigation into the negative role of EFoF, particularly focusing on practical areas like the implementation of entrepreneurial education [17,23,36]. The findings imply that EFoF might undermine the effectiveness of entrepreneurial education [17]. This echoes the critique that entrepreneurship research and education in practice should consider students' entrepreneurial psychological experiences [37,82], rather than solely prioritizing efficient business management, opportunity identification and exploitation, and leadership development.

The college students sampled in the literature were distributed worldwide, but concentrated in Asian and European countries. We posit that this distribution is related to the level of entrepreneurship activity in different regions and the political dynamics of entrepreneurship. Some social norms, cultural customs, and economic circumstances can also play critical roles in shaping college students' experiences with an EFoF [8,12]. Further empirical studies are required to confirm these findings. In addition, evidence suggests that this field lacks qualitative research to provide inductive evidence. Research needs to further explore unique factors, such as the role of parents and higher education experiences, to enrich the theoretical understanding of EFoF among college students. Notably, the current literature employs disparate measurement tools for college students' EFoF, and there exists no consensus in this regard. We identify this as another gap in the literature, where existing EFoF studies have overlooked the unique traits and backgrounds of college students in both conceptualization and measurement. Future research should consider conceptualizing college students' EFoF and developing suitable scales for this special group.

**Entrepreneurship Culture, Policies, and Regulations**. We know that entrepreneurial culture, policies, and regulations exert a lasting influence on individuals' EFoF, including high failure-tolerance policies, low failure-stigma culture, and a democratic political system [16,42,[84], [85], [86]]. However, the current entrepreneurial governance and propaganda seem to focus excessively on business operations and financial benefits, which may not fully address the unique needs and challenges faced by college students, especially the most severe psychological barriers to EFoF. Scholars should consider varying levels of development, distinctive political and economic forms, and even the prevalence of corruption in different countries and regions when analyzing this subject [55]. These factors can influence college students' perceptions of and attitudes toward entrepreneurship and failure. For instance, the “face-saving” culture in China prioritizes maintaining one's reputation and avoiding embarrassment at any cost; in this cultural context, entrepreneurial failure could be significantly harmful. While this cultural factor influences EFoF among Chinese college students, an in-depth investigation is still lacking. Similarly, Chinese collectivism may also impact college students [102]. These findings require us to further examine the direct connections between these institutional factors and college students' EFoF, as our review found that most studies did not specifically investigate the relationship, but examined related control variables. Given that college students are a unique group shielded by schools and families, their sensitivity to relevant policies does not seem to be as high as that of others.

**Entrepreneurship Education.** Our scoping review indicates that EFoF, as a form of entrepreneurial psychology among college students, is influenced by entrepreneurial education. This finding aligns with previous research, confirming the role of entrepreneurship education in mitigating individuals' EFoF [[88], [89], [90]]. Specifically, related courses and training could alleviate students' EFoF by equipping them with knowledge, skills, and a resilient mindset, enabling them to navigate challenges and confidently embrace entrepreneurial endeavors confidently [57,89,91]. Chapman and Phillips [92] further supported this notion using a large sample from the GEM, revealing that countries with more robust entrepreneurship education programs exhibit lower levels of EFoF. Future entrepreneurial curricula should incorporate EFoF-related components to help college students correctly understand entrepreneurial failure and handle psychological barriers [103]. Research can also examine the effectiveness of reformed entrepreneurship education involving curriculum design, student experience, and longitudinal tracking.

**Role Model.** The role of business models in reducing college students' EFoF conforms to the traditional understanding of role models in developmental psychology, in which role models are known to assist children in overcoming fear emotions [10]. Similar conclusions regarding the positive effects of business role models on EFoF have been confirmed by other entrepreneurial groups [10,42,87,60,91]. Exposure to successful business role models empowers college students to view failure as a regular aspect of entrepreneurship, which, in turn, encourages them to overcome EFoF, and thus foster inspiration, guidance, and a sense of possibility [10,87]. Conversely, Wyrwich et al. [60] claimed that knowing failed business celebrities increases college students' EFoF—a reminder for policymakers that potential negative consequences need to be considered when using entrepreneurial celebrity cases to promote college student entrepreneurship.

**Catastrophic Incident.** Regarding catastrophic incidents, traditional beliefs hold that they produce uncontrolled impacts and negative outcomes for business ventures. Sheng and Chen [46] indicated the favorable impact of catastrophic incidents on college students' entrepreneurial intention, which was mediated by EFoF. Therefore, officials who promote college student entrepreneurship should consider the psychological burden (e.g., EFoF) that catastrophic incidents impose on college students. Interestingly, Games et al. [62] provided a similar answer: EFoF contributes to business success in the context of an earthquake. They found that catastrophic incidents have a dualistic impact on EFoF, as such events created opportunities for innovation but also introduced uncertainties and risks. Further empirical investigation is necessary to understand the precise impact and underlying mechanisms of catastrophic incidents on college students' entrepreneurial pursuits and their experiences with EFoF.

**Gender.** Gender has been the focus of many studies on EFoF, revealing notable variations across contexts and populations, including college students [41,[93], [94], [95], [96]]. The prevailing view suggests that women tend to perceive higher EFoF levels than males. This observation aligns with the prevailing gender stereotypes in entrepreneurship, where risk-taking and a tough attitude are considered masculine traits, while women are often assigned more cautious and sensitive roles in business ventures.

Another viewpoint indicates that women exhibit greater sensitivity and willingness to express their emotions and thoughts in social roles, whereas societal expectations of masculinity discourage men from sharing their feelings openly, such as EFoF, to avoid being perceived as vulnerable [51,97]. In addition, gender, as an endogenous factor, does not innately affect college students’ EFoF. Future investigations should incorporate a broader range of sociocultural factors. For instance, societal expectations and cultural norms often prescribe traditional gender roles [64], which then influence college students' perceptions of suitable career paths. These expectations shape how male and female students view entrepreneurship and perceive the appropriateness of pursuing business ventures.

**Projected Outcomes.** Projected outcomes related to failure inevitably induce EFoF in college students. We know that projected outcomes lead students to continuously focus on the losses and shame related to business failure, thereby significantly increasing their EFoF [7,14,98]. The EFoF elicited by projected failure outcomes hinders students' further actions [48]. Cacciotti et al. [7,48] even developed six themes to measure EFoF levels. However, college students have unique social identities and backgrounds, and factors such as higher education experiences and parental career expectations may have a crucial impact on their EFoF [8]. Specifically, EFoF may be triggered by projected outcomes, where failed college student entrepreneurs lay behind in academic performance and disappointed their parents. Previous studies have not yet included these factors among college students, which calls for future research.

**Personal Traits.** The person–environment model recognizes personal traits as the endogenous factor that influences college students' entrepreneurship and EFoF, supported by a number of such studies [48,79,99]. Certain personal traits such as perfectionism, self-efficacy, resilience, optimism, and locus of control have been found to have a significant impact on individuals' risk perception, EFoF, ability to handle failure, and willingness to engage in entrepreneurship [3,4,37,104]. However, evidence on the relationship between personal traits and college students’ EFoF is insufficient owing to a lack of extensive studies on the specific personal traits exhibited by college student entrepreneurs. Future research could include traits such as adaptability, emotional intelligence, or specific learning styles and their correlation with EFoF.

**Perceived Knowledge and Skills**. Regarding the impact of perceived knowledge and skills on college students' EFoF, our findings matched those of earlier studies [82,91,99]. Potential college student entrepreneurs typically assess their abilities and available resources to determine their preparedness for starting new ventures. Perceiving themselves as possessing adequate entrepreneurship knowledge and skills enhances entrepreneurial self-efficacy, diminishing fear and concerns regarding potential failures [39]. Conversely, lower levels of perceived knowledge and skills can engender uncertainty and self-doubt, leading to elevated levels of EFoF [43,89]. These findings support the case for reforms in entrepreneurship education. Currently, entrepreneurship education primarily focuses on imparting business skills and developing leadership skills, while neglecting the psychological challenges that college students may face during their entrepreneurial journeys. Instructors should develop course content that incorporates interventions and support systems meant to enhance college students' entrepreneurial self-efficacy and address the psychological barriers that aspiring student entrepreneurs face.

## 5 Conclusion, limitations, and future research

EFoF is a significant obstacle that hinders college students from initiating their own ventures, but a clear and systematic understanding of the role of EFoF in college students’ entrepreneurship, including its influencing factors, is missing. This scoping review synthesized and evaluated 35 of the most updated studies, published between 2010 and 2023, targeting college students. The review confirmed the growing interest in researching EFoF among college students. However, we also identified a lack of qualitative exploration and use of consistent measurement tools. We also thematically analyzed the included literature and identified factors influencing college students' EFoF as well as the impact of EFoF on college students. We outlined the gaps in research identified in the literature; these include the unclear mechanism of EFoF among college students and the absence of coping strategies to handle EFoF.

With the advancement of research and practical solutions, our study lays the groundwork for the following generation of college student entrepreneurs to overcome fear and thrive in their entrepreneurial endeavors. It highlights the necessity for aspiring college student entrepreneurs, policymakers, educators, and relevant practitioners to fully grasp the concept of EFoF among college students. Acknowledging this psychological barrier enables young generations with entrepreneurial ambition to effectively handle EFoF, further enabling them to tackle challenges and seize opportunities within the entrepreneurial landscape.

Meanwhile, our study also underscores the urgency for practitioners in entrepreneurial policies, regulations, and education to reflect on and refine their workings based on our findings. For instance, future entrepreneurship education programs could develop content on entrepreneurial psychology, such as EFoF. By offering courses or workshops that simulate the entrepreneurial journey and the experience of business failure, young entrepreneurs can become more adept at recognizing and managing significant setbacks. The study also urges policymakers to develop a more supportive ecosystem for young entrepreneurs. Inspired by our findings, they can develop policies that provide better mental health support, financial safety nets, and failure recovery programs, thereby reducing fear of failure and encouraging more young generations to start businesses. By developing these focused initiatives and services, a positive and resilient entrepreneurial mindset can be nurtured among college student entrepreneurs, which is essential for the future development of entrepreneurship among young generations.

### 5.1 Limitations

This study has some limitations. First, this study exclusively included studies published in English; studies published in other languages were excluded because of limited time and budget constraints. This exclusion could result in the neglect of information regarding EFoF among college students in diverse linguistic and cultural contexts. Further research should broaden the scope by recruiting researchers from multicultural backgrounds and incorporating multilingual literature to investigate this topic.

Second, our study focused on college students' EFoF; studies with related topics or variables, such as entrepreneurial anxiety, fear, and worry, were excluded [66,105], possibly leading to a loss of insightful viewpoints. During literature mining, the descriptions of some themes (e.g., entrepreneurial anxiety) vaguely indicated a similar meaning to EFoF [7,9,48,105]. For a multidisciplinary theoretical framework of EFoF, we recommend an in-depth investigation to clarify the relationship between EFoF and other negative entrepreneurial emotions [65,101].

Finally, the scoping review paradigm does not rigorously evaluate the methodological quality of studies, which a systematic review does. Nevertheless, this scoping review will aid future researchers in gaining a broad and clear understanding of EFoF research among college students, and also serve as a reference for subsequent systematic review [28].

### 5.2 Future research

Building upon this scoping review, future studies should address four significant research gaps to advance our understanding of EFoF among college students. First, we recommend examining the impact of factors arising from college students’ unique characteristics and backgrounds on their EFoF, such as parental roles and higher education experiences. We know that parental support can weaken EFoF in college students; however, in the Chinese context, parents encultured by a conservative ethos prefer that their children choose stable jobs, such as civil servants [6], rather than riskier endeavors such as entrepreneurship. It is reasonable to assume that disagreements among Chinese parents may predict their EFoF, a point that requires further examination.

Second, entrepreneurial passion is a notable trait among college student entrepreneurs, and it positively influences entrepreneurial intention and self-efficacy [106]. Considering the adverse impact of EFoF on college student entrepreneurship [17,18], it may be negatively moderated; however, further testing is needed.

Third, the understanding of EFoF among college students remains incomplete and is subject to debate, given that most of the evidence is derived from cross-sectional and quantitative studies. Specifically, college students experience a transition from students on campus and children in the family to marketing [20,21]. This transition entails the recognition of social identities, responsibilities, and attitudes toward failure, as students navigate the complex landscape of entrepreneurship [21]. Future qualitative studies should employ in-depth interviews or focus groups to establish a comprehensive conceptual framework and explore the underlying mechanisms of EFoF among college students. Longitudinal research can also be a valuable strategy for monitoring changes in EFoF among college students during emerging adulthood, if capacity allows.

Fourth, we also suggest adapting or developing measurement instruments specific to college students, given their distinctive social identities and contextual circumstances. As stated earlier, there is no consensus on how to empirically measure EFoF among college students. The measurement tools currently in use were not originally developed for samples of college students. We recommend first operationalizing the definition of entrepreneurship with respect to college students, followed by inductively constructing a conceptual framework, and then developing new or modifying existing scales. We believe this methodical approach to measurement would ensure the reliability and validity of college students’ EFoF assessments.

Finally, there exists a dearth of experimental research on strategies and procedures for addressing EFoF among college students. We hope to see intervention programs and development initiatives established that are based on a clear understanding of the nature and mechanism of EFoF. These efforts should provide solutions and benefits to various stakeholders, including college student entrepreneurs, parents, schools, and governments.

## Declaration

This study was approved by the Ethics Committee of Xi'an Jiaotong-Liverpool University (Application Number: 0010000100120220802153931). The authors declare no conflicts of interest. All authors listed have contributed significantly to the development and writing of this article. This study did not receive any specific grants from funding agencies in the public, commercial, or non-profit sectors.

## Data availability statement

Data associated with this scoping review have not been deposited in any public repository. All data used in this article have been referenced herein.

## CRediT authorship contribution statement

**Yuan Gao:** Writing – review & editing, Writing – original draft, Methodology, Formal analysis, Data curation, Conceptualization. **Xiao Wang:** Writing – review & editing, Methodology. **Jinjin Lu:** Writing – review & editing, Supervision, Methodology. **Bing Chen:** Writing – review & editing, Supervision. **Kirsty Morrin:** Writing – review & editing, Supervision.

## Declaration of competing interest

The authors declare that they have no known competing financial interests or personal relationships that could have appeared to influence the work reported in this paper.
