# Supplementary material for: Entrepreneurial fear of failure among college students: A scoping review of literature from 2010 to 2023
Source: Heliyon. 2024 May 11;10(10):e31072. doi: 10.1016/j.heliyon.2024.e31072 (PMC11109765; doi:10.1016/j.heliyon.2024.e31072)
Supplement: Multimedia component 2 [file mmc2.docx]

**Entrepreneurial fear of failure among college students: A scoping review of literature from 2010 to 2023**

# 6. Appendix

| **Appendix 1**  *Descriptive Summary of Studies included in This Scoping Review* | | | | | | |
| --- | --- | --- | --- | --- | --- | --- |
| Author (Year) | Research Aim(s) | Perspective on EFoF | Design | participants | Measurement | Key Finding(s) |
| Ng and Jenkins (2018) | To understand different aspects of fear of failure impact the relationship between entrepreneurial self- efficacy and entrepreneurial intentions. | A dispositional tendency to experience apprehension and anxiety upon failure | Quantitative, cross-sectional, regression analysis | 182 Third-Year university students in business management in Australia | Performance Failure Appraisal Inventory (PFAI) by Conroy et al. [45] | Fear of failure was one mechanism which dampened the positive relationship between entrepreneurial self-efficacy and entrepreneurial intentions. |
| Chua and Bedford (2016) | To investigate the construct of fear of failure in the context of entrepreneurial intent in Singapore. | A temporary emotional state | Qualitative, cross-sectional, semi-structured interview | 35 third- and fourth-year undergraduate students in diverse majors in Singapore | Interview guide | Three key themes associated with the perceived negative impact of entrepreneurial failure were revealed: financial, psychological, and career. |
| Kong et al. (2016) | To examine the moderating effect of business role models and fear of failure on the relationship between entrepreneurial intention and behavior. | Risk aversion, an obstacle to their entrepreneurship | Quantitative, hierarchical regression | 698 university students in China | One dichotomous question for measuring fear of failure | Fear of failure hindered college students from taking entrepreneurial behaviors, and business role models would enhance their entrepreneurial intention. |
| Duong (2022) | To examine the moderating effects of entrepreneurial fear of failure on the link between the entrepreneurial intention and behavior, and its effects on the mediation effect of attitude towards entrepreneurship on entrepreneurial behavior through intention. | A significant psychological barrier to entrepreneurial activities | Quantitative, cross-sectional, hierarchical regression | 611 master degree students in Vietnam | Entrepreneurial Fear of Failure Scale by Cacciotti et al. [9] | Entrepreneurial attitude mediated the link between entrepreneurial attitude and behavior. Entrepreneurial fear of failure negatively moderated the link between entrepreneurial intention and behavior. |
| Li (2011) | To investigate the effects of anticipated emotions on individuals’ judgment on value and probability of entrepreneurship. | Negative emotion with risk aversion | Quantitative, cross-sectional, hierarchical regression | 217 undergraduate students in business school with Chinese ethnicity | Simulation Heuristic Measurement | Individuals' anticipated emotions affected value and probability judgment of a new venture. Those who showed less fear of failure and lower surprise for the success tended to view a new venture as an opportunity. |
| Shinnar et al. (2012) | To examine how culture and gender shape entrepreneurial perceptions and intentions within Hofstede's cultural dimensions framework and gender role theory. | Risk aversion | Quantitative, cross-sectional, partial least squares (PLS) approach to structural equation modeling (SEM) | 761 University students (147 Chinese, 285 American, and 329 Belgian) | One dichotomous question for measuring fear of failure | Culture and gender did matter when it came to perceptions of barriers to entrepreneurship and their relationship with entrepreneurial intentions. Fear of failure was a more important barrier for Women in America and Belgium than men, but it did not exist in China. |
| Martins et al. (2018) | To identify the impact of personality features, such as self-confidence and fear of failure, on the entrepreneurial orientation of university students enrolled on entrepreneurial education courses. | A barrier for entrepreneurial activity | Quantitative, cross-sectional, linear regression | 656 college students in Colombia | One dichotomous question for measuring fear of failure | Both self-confidence and fear of failure were determinants of the entrepreneurial orientations of university students. Fear of failure had a negative effect on their entrepreneurial orientations, including all three dimensions: risk-taking, innovativeness and proactiveness. |
| Singh Sandhu et al. (2011) | To examine the relationship between perceived barriers (fear of failure) to entrepreneurship and entrepreneurial inclination among postgraduate students. | A barrier for entrepreneurial activity | Quantitative, cross-sectional, multiple regression | 267 postgraduate students in Malaysia | Adjusted five-item scale by Scott and Twomey [76] | Fear of failure was an important obstacle for graduate students' entrepreneurial inclination, together with other perceived barriers. |
| Kollmann et al. (2017) | To demonstrate the responsiveness of fear of failure to situational cues in an entrepreneurship setting (study 1). | A responsive avoidance motive | Quantitative, cross-sectional, experimental approach, regression | 71 university students enrolled on entrepreneurship course in German | Revised Achievement Motives Scale (AMS) by Lang and Fries [47] | In Study 1, fear of failure was responsive to (i.e., activatable by) failure-relevant situational cues in an entrepreneurship setting and consequently affected entrepreneurial decision-making. |
| Turulja et al. (2020) | To analyze the influence of different kinds of support (perceived formal, informal and regulatory support) on the entrepreneurial intention of students in Bosnia and Herzegovina. | A psychological barrier to entrepreneurship | Quantitative, cross-sectional, structural equation modeling | 111 college students in Economics and Business in Bosnia and Herzegovina | One dichotomous question for measuring fear of failure | Fear of failure was a significant negative predictor of entrepreneurial intention. Informal support moderated the relationship between fear of failure and entrepreneurial intention. |
| Iancu et al. (2021) | To understand the factors that influence the college students' intention to start new business in Romania. | A barrier for entrepreneurship | Quantitative, cross-sectional | 300 undergraduate and postgraduate students in Economics and Business in Romania | One dichotomous question for measuring fear of failure | Fear of failure significantly diminished the entrepreneurial intention among college students in Romania. |
| Urbano et al. (2017) | To investigate the influence of informal and formal institutions on the university students' decision of entrepreneurship in Catalonia (Spain). | A barrier for entrepreneurship | Quantitative, probit regression | 1207 college students from Catalonia in Spain | One dichotomous question for measuring fear of failure | Fear of failure (informal institution) had a negative effect on the probability of university students becoming intrapreneurs. |
| Meeralam and Adeinat (2022) | To examine the impact of several personal, cultural and social antecedents on female students’ intentions to become entrepreneurs. | A state inhibiting the transformation from entrepreneurial intention into activity | Quantitative, cross-sectional, structural equation modeling | 740 female college students in Saudi Arabia | Self-adjusted five-item scale | Overcoming fear of failure had a significant positive effect on female students' attitude toward entrepreneurship and indirectly affected their entrepreneurial intention. |
| Thoudam et al. (2022) | To measure the direct and moderated influence of entrepreneurial passion, motivation and creativity on intention while being moderated by entrepreneurship education. | A psychological barrier to entrepreneurship | Quantitative, cross-sectional, structural equation modeling and Process Macro | 1090 undergraduate and postgraduate students from five universities in India | One dichotomous question for measuring fear of failure | Entrepreneurial passion, motivation and creativity could directly affect entrepreneurial intention, and entrepreneurial education moderated these links; and fear of failure moderated those moderated paths among entrepreneurial education, passion, motivation, creativity and intention. |
| Hassan et al (2022) | To explore the influence of psychological and contextual variables (fear of failure) in shaping entrepreneurial motivations and the subsequent formation of entrepreneurial intention among college students. | A constraint to entrepreneurial motivation | Quantitative, cross-sectional, structural equation modeling | 329 college students who received entrepreneurship education before in India | Entrepreneurial Fear of Failure Scale by Cacciotti et al. [9] | Fear of failure had a significant impact on individuals' entrepreneurial motivations. Contextual factors of government support policies and access to entrepreneurial finance did not influence entrepreneurial motivations. |
| Villanueva and Martins (2022) | To verify whether overconfidence, fear of failure, risk- taking, and risk capacity affect overall risk evaluation. | An obstacle to entrepreneurship | Quantitative, cross-sectional, hierarchical regression | 828 undergraduate students in different courses in Colombia | One dichotomous question for measuring fear of failure | Fear of failure did not directly affect undergraduate students' entrepreneurial intentions, but it was positively and significantly related to their risk evaluation. |
| Ukil and Jenkins (2022) | To understand how fear of failure and resilience influence college students' entrepreneurial intentions, within the context of low job prospects and high unemployment rate. | A mental barrier, inhibiting entrepreneurial behavior | Quantitative, cross-sectional, structural equation modeling | 238 third and fourth-year college students in Bangladesh | Adjusted short scale from the Performance Failure Appraisal Inventory (PFAI) by Conroy et al. [45] | Fear of failure decreased college students' entrepreneurial intentions. Resilience had a positive influence on their entrepreneurial intentions, and decreased the negative influence from fear of failure. |
| Nefzi (2018) | To examine the impact of fear on entrepreneurial risk perception taking into account the mediation effects of certainty and controllability. | Both state and trait, a barrier to new venture creation | Quantitative, cross-sectional, multivariate analysis of covariance and structural equation modeling | 63 undergraduate students in entrepreneurship course in Tunisia | Performance Failure Appraisal Inventory (PFAI) by Conroy et al. [45]. | Fear of failure (state) did not intensify the level of entrepreneurial risk through uncontrollability; Fear of failure (trait) indirectly aroused the entrepreneurial risk perception through their impacts on uncertainty. |
| Sheng and Chen (2022) | To investigate the effect of event strength (COVID-19) on college students' entrepreneurial intentions, alongside the mediating roles played by promotion regulatory focus and fear of failure and prevention regulatory focus and fear of failure. | Fearful emotions caused by perceived environmental threats | Quantitative, cross-sectional, hierarchical regression | 245 college students in China | Revised Achievement Motives Scale (AMS) by Lang and Fries [47] | The event strength of COVID-19 pandemic had a positive effect on college students' entrepreneurial intentions and this relationship could be transmitted through the continuous mediating role of regulatory focus and fear of failure. |
| Voegel and Voefel (2019) | To examine the relationships between an individual's gender identity (whether they identify as masculine or feminine) and their entrepreneurial intention. | A barrier for entrepreneurship | Quantitative, cross-sectional, structural equation modeling | 378 college students in United States | One dichotomous question for measuring fear of failure | Gender identity affected the individual's perceived fear of failure. Individual with feminine identity would exhibit higher levels of perceived fear of failure. Fear of failure was a significant barrier for entrepreneurial intention. |
| Belwal et al. (2015) | To investigate students' perceptions of entrepreneurship and the role of universities in developing enterprise education in Oman, focusing on the case of Sohar University. | A barrier for entrepreneurial entry | A mixed method approach, cross-sectional, | 200 university students from different majors in Oman | Not clearly stated | Most of the university students were optimistic and interested in entrepreneurship, but lacked knowledge about how to start a business. Fear of failure and unwillingness to take risks were the major obstacles facing university students in treading an entrepreneurial path. |
| Sánchez Cañizares and Fuentes García (2010) | To analyze the role of gender differences among potential entrepreneurs, their psycho-sociological traits and the incentives and principal obstacles women encounter when initiating a business activity. | A barrier for entrepreneurship | A mixed method approach, cross-sectional, logistic regression | 1400 university students in Spain | Self-adjusted  instrument. | The entrepreneurial initiative was lower among female students, and they were more likely to view the fear of failure as an obstacle for entrepreneurship than men. |
| Kateřina and Jana (2019) | To investigate the most prominent entrepreneurial motives and barriers, and gender differences in entrepreneurship among university students. | A barrier for entrepreneurial entry | Quantitative, cross-sectional, multivariate analysis of covariance and U Test | 1021 third-and-fourth year college students from diverse majors in Czech Republic | Self-developed questionnaire | Besides lack of financial resources and complex bureaucracy, fear of failure was one of the most important barriers for entrepreneurship. |
| Kebaili et al. (2015) | To explore the major entrepreneurship barriers facing Qataris. | The negative reaction of the society to failed entrepreneurs | Qualitative, cross-sectional, semi-structured interview | 18 final year business students in Qatar | Interview guide | Seven factors were identified as the main barriers to start-up new business activities among Qataris, including the non-availability of funds, risk avoidance, fear of failure, market barriers, knowledge barriers, stress avoidance, and attitude towards change. |
| Ekore and Okekeocha (2012) | To examine the role of psychological factors, especially fear of failure as an entrepreneur, among university graduates, in this case in Nigeria. | A barrier for entrepreneurship | Quantitative, cross-sectional, multiple regression | 1100 graduate students in Nigeria | Self-developed instrument with 24 items | College students with positive entrepreneurial attitude had less fear of failure while those with negative attitude showed more fear. College students with higher perceived entrepreneurial capacity showed less fear of failure. |
| Salavou and Lioukas (2019) | To investigate capability and vulnerability self-images among three groups of entrepreneurs to understand differences in perceptions. | Related to vulnerability self-images | Quantitative, cross-sectional, logistic regression | 617 graduate entrepreneurs in Greece | Self-adjusted ten-item scale. | Regarding vulnerability self-images, fear was shared among all kinds of entrepreneurs. Nascent entrepreneurs experienced more fears than actual entrepreneurs. High-intention nascent entrepreneurs were less afraid of failing than low-intention ones. |
| Anwar et al. (2022) | To build on psychological and contextual determinants of entrepreneurial intention and gather primary data using questionnaires. | Psychological factor influencing entrepreneurial intention | Quantitative, cross-sectional | 329 undergraduate and postgraduate level students in business and management courses in India | Adjusted scale from Cacciotti et al. [9] | The research provided the primary survey-based data to understand the cognitive and psychological factors of college student entrepreneurship in India. Also, the study provided multidimensional data on fear of failure. |
| Smail et al. (2022) | To investigate the determinants of entrepreneurial risk among Emirati youth enrolled in a university in the United Arab Emirates. | A barrier for entrepreneurship | Quantitative, cross-sectional, ordinal probit regression estimation and structural equation modeling | 324 university students in the United Arab Emirates | Self-adjusted scale. | Fear of failure positively related to risk aversion. College students in the research were more willing to bear uncertainties, and it was possibly attributed to the favorable and encouraging government support. Entrepreneurship education alleviated college students' fear of failure. |
| Soomro et al. (2022) | To investigate the significant barriers that Pakistani Female entrepreneurs need to overcome. | A barrier for entrepreneurship | Quantitative, cross-sectional, structural equation modeling | 498 students in bachelor and master programs in Pakistan | Five-item scale from Singh Sandhu et al. [32] | Fear of failure had a negative effect on entrepreneurial intention of Pakistani female college students. Fear of failure reduced their confidence at being successful in business, and affected their workplace performance. |
| Ruda et al. (2012) | To analyze the differentiated entrepreneurial tendencies and characteristics of university students from different countries, and propose actions for demand-oriented entrepreneurship education. | A barrier for entrepreneurship | Quantitative, cross-sectional | 3500 university students in Germany, and 400 university students in Russia | Not clearly stated | The risk of failure could induce serious consequences, especially in high-tech sectors. The students in Germany regarded their own financial risk and fear of failure as higher start-up barriers than those in Russia. |
| Sihaloho et al. (2021) | To explore the causes of fear of failure in entrepreneurship experienced by college students along with things that affect their entrepreneurial fear of failure. | A barrier for entrepreneurship | Qualitative, literature review method | Scientific journals and articles that have official ISSN | Not clearly stated | Higher education students’ entrepreneurial fear of failure was caused by financial consequences and psychological crises. |
| Hussaini (2019) | To investigate the impact of financial management expertise on entrepreneurship development among the students of Kuwait. | A barrier for entrepreneurship | Quantitative, cross-sectional, regression | 305 university students in Kuwait | Not clearly stated | Among the influential factors/barriers, bad experience of others and educational background, government policies and fear of failure had their substantial influence on the college students' attitude towards entrepreneurship activities. |
| Duong and Vu (2023) | To investigate the moderating impacts of entrepreneurial fear of failure and gender on the direct and mediation relationships between entrepreneurial education, entrepreneurial self-efficacy and entrepreneurial intention. | A significant psychological barrier to entrepreneurial activities | Quantitative, cross-sectional | 1890 master students in Vietnam | Entrepreneurial Fear of Failure Scale by Cacciotti et al. [9] | Entrepreneurial fear of failure negatively moderated the impacts of Entrepreneurial education on both entrepreneurial self-efficacy and entrepreneurial education. Entrepreneurial fear of failure significantly moderated the indirect effect of entrepreneurial education on entrepreneurial intention through entrepreneurial self-efficacy. |
| Sousa-Filho et al. (2023) | To develop a deeper understanding of the motivational processes involved in entrepreneurial behavior intent, and it integrates fear and entrepreneurship into the Theory of Planned Behavior. | A significant obstacle to various entrepreneurial activities | Quantitative, cross-sectional, Structural equation Modelling | 979 college students in business from Latin America (Brazil, Peru, Columbia, Mexico) | Performance Failure Appraisal Inventory (PFAI) by Conroy et al. [45] | Entrepreneurial fear of failure affected attitude and perceived behavior control negatively. Study includes entrepreneurial fear of failure as an antecedent of entrepreneurial attitude and perceived behavior control. |
| Shukla (2023) | To investigate the perspective of Graduate and Post-graduate students of the university towards entrepreneurship as a career path. | The biggest factor preventing most people from beginning their own enterprises. | Quantitative, cross-sectional | 500 college students in India | Self-developed questionnaire | Entrepreneurial fear of failure has a negative influence on entrepreneurial inclination of the students. |
